# Inguinal Ulceroglandular Tularemia Caused by *Francisella tularensis* Subspecies *holarctica*, Canada

**DOI:** 10.3201/eid2704.203262

**Published:** 2021-04

**Authors:** Carl Boodman, Quinlan Richert, Sylvain Lother, Ken Kasper, Sergio Fanella, Philippe Lagacé-Wiens, Yoav Keynan

**Affiliations:** University of Manitoba, Winnipeg, Manitoba, Canada; Shared Health Inc., Winnipeg (P. Lagacé-Wiens)

**Keywords:** tularemia, vector-borne infections, genital ulcer disease, *Francisella tularensis*, laboratory-acquired infection, bacteria, Canada, ticks, tick-borne infections

## Abstract

Tularemia is a zoonotic disease caused by the gram-negative coccobacillus *Francisella tularensis,* a Biosafety Level 3 pathogen and potential agent of bioterrorism. We describe 2 cases of perigenital ulcer disease caused by *Francisella tularensis* subspecies *holarctica* in Manitoba, Canada. These cases caused inadvertent exposure among laboratory personnel.

In July 2018, a previously healthy girl 4 years of age was brought to the Health Sciences Centre at University of Manitoba (Winnipeg, Manitoba, Canada) for fever, right inguinal swelling, and dysuria. The patient’s symptoms had worsened despite completing a 5-day course of trimethoprim/sulfamethoxazole prescribed by her family doctor for a presumed urinary tract infection 1 week before admission. The patient lived on a rural property bordered by forest in southern Manitoba, Canada. She enjoyed playing with her dogs and cats and often returned from the yard with ticks embedded in her skin.

At admission, her vital signs were within normal limits. We noted a small ulcer lateral to the right labia majora. This shallow nonpurulent ulcer was <1 cm long, surrounded by erythema, and accompanied by tender local lymphadenopathy. We took a swab sample of the ulcer for bacterial culture and prescribed ceftriaxone for presumed cellulitis.

Three days after admission, the culture revealed pinpoint growth of *Francisella tularensis* on chocolate agar, identified by matrix-assisted laser desorption/ionization-time of flight mass spectrometry (Bruker, https://www.bruker.com) (*1*). However, because a Biosafety Level 3 (BSL-3) pathogen had not been suspected, the culture was manipulated outside a biosafety cabinet (BSC). The exposed laboratory technologist was prescribed oral doxycycline (100 mg 2×/d for 14 days) as postexposure prophylaxis; the technician showed no signs or symptoms of tularemia. The bacterial isolate was classified as a UN2814, category A infectious substance; it was mailed in a sealed container with polystyrene foam-insulated packaging and an established Emergency Response Assistance Plan and placed in a box displaying the biohazard symbol to Canada’s National Microbiology Laboratory (NML) for subspeciation. The NML identified the isolate as *Francisella tularensis* type B subspecies *holarctica*. We treated the patient for ulceroglandular tularemia (20 mg/kg of oral ciprofloxacin 2×/d for 14 days), prompting a complete recovery.

In August 2019, a woman 60 years of age arrived at Brandon Regional Health Centre (Brandon, Manitoba, Canada) with acute onset of hypotension and an ulcer beside her right labia majora. She had had chills for several days before seeking care. She had end-stage renal disease managed by hemodialysis and sick sinus syndrome managed by a pacemaker. The patient lived in a rural area of southern Manitoba and had found a tick attached to her abdomen ≈1 week before admission. She was not sure how long the tick had been attached; she removed it upon discovery.

At admission, the patient was hypotensive (70/25 mm Hg). She had a paced heart rate of 60 bpm and oxygen saturation of 99% on room air. She did not have a fever. We noted a 2 cm long ulcer beside the right labia majora with surrounding erythema and bilateral inguinal lymphadenopathy. We found a 2 cm long necrotic eschar with surrounding erythema at the site of tick attachment.

The gram stain cultured from the perivulvar ulcer showed no organisms. However, faint growth appeared on chocolate agar on day 3. We identified *F. tularensis* using matrix-assisted laser desorption/ionization-time of flight mass spectrometry (*1*). Before speciation, the culture had been manipulated outside a BSC, resulting in the exposure of 1 technologist; this technologist received doxycycline for 14 days as prophylaxis and had no signs or symptoms of infection. The NML identified the sample as *F. tularensis* subspecies *holarctica*. The patient was treated with gentamicin (2 mg/kg 1×/d for 7 days) and oral ciprofloxacin (500 mg 1×/d for 14 days) and symptoms resolved. We did not conduct serologic tests on samples from either patient. We notified the medical health officer of both cases.

The low infectious dose and easy dissemination of *F. tularensis* pose a substantial risk for laboratory-acquired infections when manipulated outside of a BSC (*2*,*3*). The perigenital localization of tularemia in these cases produced an especially hazardous situation for laboratory exposure; in contrast to blood, lymph node, and bone marrow samples, genital lesions are not usually suspected to harbor BSL-3 pathogens. A history of animal or arthropod exposure is a risk factor that can alert laboratory staff to the possibility of tularemia, enabling the application of appropriate precautions (*4*). Pinpoint colonies of gram-negative coccobacilli growing aerobically on chocolate agar 48 hours after plating might indicate the presence of *F. tularensis* and should prompt BSL-3 precautions, as emphasized by the Centers for Disease Control and Prevention’s Laboratory Response Network in affiliation with the American Society for Microbiology (*5*,*6*). Of 42 cases of laboratory-acquired tularemia documented by Overholt et al. (*7*), 16 were unsuspected by microbiologists and occurred outside of a known exposure. 

These 2 cases caused by *F. tularensis* subspecies *holarctica* support veterinary studies suggesting that this subspecies might be more common in the Canadian prairies than the more virulent *F. tularensis* subspecies *tularensis* identified elsewhere in North America (*8*–*10*). The milder symptoms associated with *F. tularensis* subspecies *holarctica* might require a higher index of clinical suspicion, especially among patients with exposure to arthropods or wild mammals.

## References

[1] Rudrik JT, Soehnlen MK, Perry MJ, Sullivan MM, Reiter-Kintz W, Lee PA, et al. Safety and accuracy of matrix-assisted laser desorption ionization–time of flight mass spectrometry for identification of highly pathogenic organisms. J Clin Microbiol. 2017;55:3513–29. 10.1128/JCM.01023-17 29021156 PMC5703816

[2] Shapiro DS, Schwartz DR. Exposure of laboratory workers to *Francisella tularensis* despite a bioterrorism procedure. J Clin Microbiol. 2002;40:2278–81. 10.1128/JCM.40.6.2278-2281.2002 12037110 PMC130659

[3] Centers for Disease Control and Prevention. Managing potential laboratory exposures to *Francisella tularensis*. 2018 [cited 2020 Oct 04]. https://www.cdc.gov/tularemia/laboratoryexposure/index.html

[4] Singh K. Laboratory-acquired infections. Clin Infect Dis. 2009;49:142–7. 10.1086/599104 19480580 PMC7107998

[5] Miller JM, Astles R, Baszler T, Chapin K, Carey R, Garcia L, et al. Guidelines for safe work practices in human and animal medical diagnostic laboratories: recommendations of a CDC-convened, biosafety Blue Ribbon Panel. [Erratum in: MMWR Surveill Summ. 2012;61:214]. MMWR Suppl. 2012;61:1–102. 10.1086/599104 22217667

[6] Craft D, Kijek T. Sentinel level clinical laboratory guidelines for suspected agents of bioterrorism and emerging infectious diseases: *Francisella tularensis*. 2016 [cited 2020 Oct 3]. https://asm.org/ASM/media/Policy-and-Advocacy/LRN/Sentinel%20Files/tularemia.pdf

[7] Overholt EL, Tigertt WD, Kadull PJ, Ward MK, Charkes ND, Rene RM, et al. An analysis of forty-two cases of laboratory-acquired tularemia. Treatment with broad spectrum antibiotics. Am J Med. 1961;30:785–806. 10.1016/0002-9343(61)90214-5 13731776

[8] Wobeser G, Ngeleka M, Appleyard G, Bryden L, Mulvey MR. Tularemia in deer mice (*peromyscus maniculatus*) during a population irruption in Saskatchewan, Canada. J Wildl Dis. 2007;43:23–31. 10.7589/0090-3558-43.1.23 17347390

[9] Wobeser G, Campbell GD, Dallaire A, McBurney S. Tularemia, plague, yersiniosis, and Tyzzer’s disease in wild rodents and lagomorphs in Canada: a review. Can Vet J. 2009;50:1251–6. 20190973 PMC2777287

[10] Antonation KS, Bekal S, Côté G, Dallaire A, Corbett CR. Multiple-locus variable-number tandem-repeat analysis of *Francisella tularensis* from Quebec, Canada. Lett Appl Microbiol. 2015;60:328–33. 10.1111/lam.12371 25442329

